# Identifying dietary patterns across age, educational level and physical activity level in a cross-sectional study: the Tromsø Study 2015 - 2016

**DOI:** 10.1186/s40795-022-00599-4

**Published:** 2022-09-15

**Authors:** Åse Mari Moe, Sigrunn H. Sørbye, Laila A. Hopstock, Monica H. Carlsen, Ola Løvsletten, Elinor Ytterstad

**Affiliations:** 1 Department of Mathematics and Statistics, UiT The Arctic University of Norway, Tromsø, Norway; 2 Department of Community Medicine, UiT The Arctic University of Norway, Tromsø, Norway; 3 Division of Nutritional Epidemiology, University of Oslo, Oslo, Norway

**Keywords:** Diet groups, Dietary patterns, FFQ, Hierarchical cluster analysis, Population studies

## Abstract

**Background:**

A healthy diet can decrease the risk of several lifestyle diseases. From studying the health effects of single foods, research now focuses on examining complete diets and dietary patterns reflecting the combined intake of different foods. The main goals of the current study were to identify dietary patterns and then investigate how these differ in terms of sex, age, educational level and physical activity level (PAL) in a general Nordic population.

**Methods:**

We used data from the seventh survey of the population-based Tromsø Study in Norway, conducted in 2015-2016. The study included 21,083 participants aged $40-99$ years, of which $72\%$ completed a comprehensive food frequency questionnaire (FFQ). After exclusion, the study sample included 10,899 participants with valid FFQ data. First, to cluster food variables, the participants were partitioned in homogeneous cohorts according to sex, age, educational level and PAL. Non-overlapping diet groups were then identified using repeated hierarchical cluster analysis on the food variables. Second, average standardized diet intake scores were calculated for all individuals for each diet group. The individual diet (intake) scores were then modelled in terms of age, education and PAL using regression models. Differences in diet scores according to education and PAL were investigated by pairwise hypothesis tests, controlling the nominal significance level using Tukey’s method.

**Results:**

The cluster analysis revealed three dietary patterns, here named the Meat and Sweets diet, the Traditional diet, and the Plant-based- and Tea diet. Women had a lower intake of the Traditional diet and a higher preference for the Plant-based- and Tea diet compared to men. Preference for the Meat and Sweets diet and Traditional diet showed significant negative and positive trends as function of age, respectively. Adjusting for age, the group having high education and high PAL compared favourably with the group having low education and low PAL, having a significant lower intake of the Meat and Sweets and the Traditional diets and a significant higher intake of the Plant-based- and Tea diet.

**Conclusions:**

Three dietary patterns (Meat and Sweets, Traditional, and Plant-based- and Tea) were found by repeated clustering of randomly sampled homogeneous cohorts of individuals. Diet preferences depended significantly on sex, age, education and PAL, showing a more unhealthy dietary pattern with lower age, low education and low PAL.

**Supplementary Information:**

The online version contains supplementary material available at 10.1186/s40795-022-00599-4.

## Background

Nutrition plays a critical role in the well-being and development of all human beings. An unhealthy diet can have severe consequences and contribute to lifestyle related conditions such as obesity, cardiovascular diseases, type 2 diabetes and cancer [1–3]. This requires a strong focus on promoting healthy diets to decrease individual risk of lifestyle diseases [4].

During the last few decades, research focus has switched from studying the health effects related to intake of single foods and nutrients to studying dietary patterns which reflect the combined intake of different foods and nutrients. A plant-based dietary pattern might reduce the risk of certain chronic diseases, while a dietary pattern high in red meet and added sugar, can potentially increase this risk [2, 5, 6]. A focus on the overall diet, rather than single nutrients, have the practical benefit of giving individuals flexibility in adapting to a healthier diet, avoiding strict nutrition advice [7].

Eating patterns vary across age, culture, lifestyle and socioeconomic status. Preferences for a healthier diet have been seen to increase with age [8–10] while diets high in meat and sweets are most prevalent among the younger population [11, 12]. A healthier diet is typically positively associated with lifestyle factors like physical activity level and educational level [11]. Specifically, higher education have been observed to give higher compliance with the national recommendations [13]. Differences in eating habits between different socioeconomic groups can contribute to inequalities in health. Investigation of dietary patterns across age and population groups is therefore important to understand and target preventive health measures.

Data used to study dietary preferences are commonly collected retrospectively by food frequency questionnaires (FFQ), in which individual intake values for a wide range of food items, dishes and beverages can be aggregated into groups of food variables. Such data can be analysed using a variety of statistical methods, see the recent review by Zhao et al. [14]. These methods include investigator-based a priori approaches, which are based on predefined diet quality scores for different food or nutrition items [15]. A posteriori approaches include classical data-driven methods for dimension reduction like principal component analysis, factor analysis and clustering [16–18].

The first aim of this study was to identify and describe dietary patterns in the seventh survey of the Tromsø Study. This is a comprehensive community based health survey situated in the north of Norway, and should reflect a general Nordic population. Dietary data was collected using a validated FFQ and robust dietary pattern analyses were conducted. A second important objective was to investigate whether there were any associations between the intakes of different diet patterns and age, sex, educational level and PAL in this community survey.

## Methods

The Tromsø Study is a population-based study conducted in the municipality of Tromsø, Norway. Seven surveys have been conducted between 1974 and 2016 (Tromsø1-Tromsø7) to which total birth cohorts and random population samples have been invited [19].

### Study sample

**Fig. 1:**
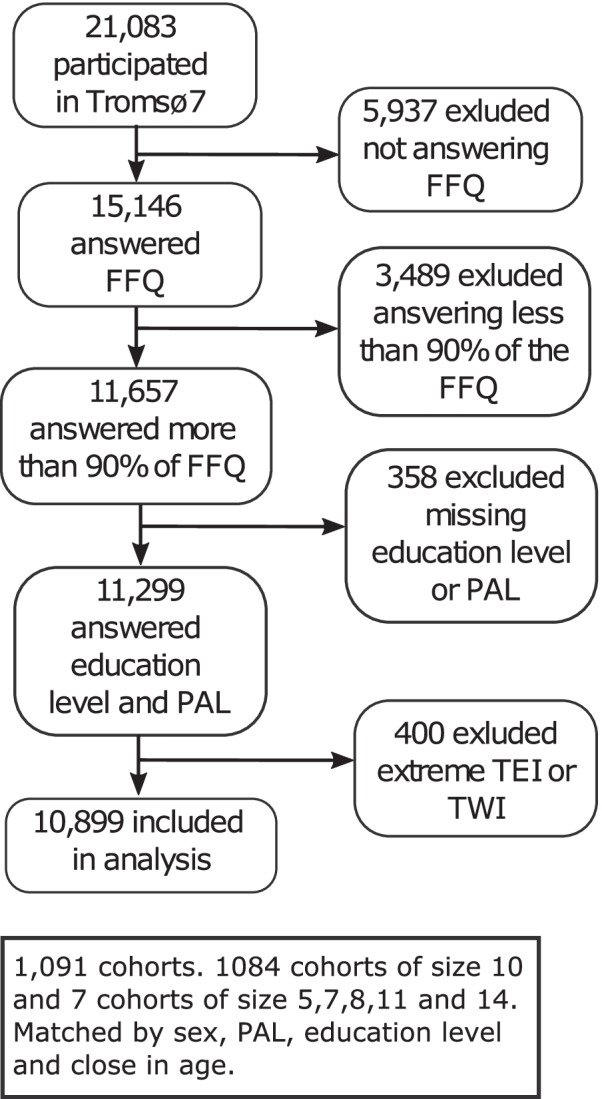
Flowchart of the study sample. FFQ: food frequency questionnaire

The present study used data from the seventh survey (Tromsø7), conducted in 2015-2016. All inhabitants of Tromsø municipality aged 40 years or above were invited and 21,083 women and men attended (65% attendance). All of the participants received an FFQ to complete on paper and to be returned by postal mail in a pre-paid envelope. The study sample comprised those who answered the FFQ ($n={15,146}$). We excluded those who completed less than 90% of the FFQ ($n={3,489}$), those with missing values on educational level or PAL ($n=358$) and those with extreme values of total energy intake or total water intake ($n=400$), see details in Additional file 1. The final study sample then comprised 10,899 participants (Fig. 1).

### Measurements

Self-reported educational level was dichotomized as Low (including primary/partly secondary with up to 9 years of schooling, and upper secondary with 10-12 years of schooling) or High (short tertiary with $<4$ years college/university and long tertiary with $\ge 4$ years college/university). Self-reported leisure-time PAL was dichotomized as Low or High according to the validated Saltin-Grimby questionnaire [20]. Specifically, the category of low PAL here includes both sedentary and light exercise, ranging from those who are almost completely inactive to those who do light physical activity at least 4 hours a week (Level 1 and 2 of the original Saltin-Grimby scale). High PAL ranges from regular and moderate training at least 4 hours a week to vigorous hard training for competitions (level 3 and 4 of the original Saltin-Grimby scale).

The FFQ, validated by Carlsen et al. [21], included questions about frequency and amount of intake of 244 food items, dishes and beverages. Calculation of total energy- (TEI), total water- (TWI), food- and nutrient intakes in kilojoule (kJ), and grams (g) per day, respectively, was performed using the food composition database and nutrient calculation system KBS at the University of Oslo, database AE14 (based on the Norwegian food composition tables 2014 and 2015), software version 7.3 [22].

We aggregated the intakes from the original 244 food items into 33 new variables plus one variable not used in this study. The aggregation was done in a supervised manner, each variable representing the total intake for groups of solid food and beverages (see Table S1, Additional file 2). This type of aggregation is in coherence with literature, in which questions of FFQs are typically summarized by 30-60 variables, see e.g. Karageorgou et al. [17].

### Preprocessing: Scaled intake values and partition of the study sample in cohorts

To identify similarities in dietary intake and the composition of food preferences, each food variable was scaled according to the individual energy intake. The scaled intake values were calculated by

$$\begin{aligned} \mathrm {Food}^*_{ji} = \mathrm {Food}_{ji}\frac{\mathrm {\overline{TEI}}}{\mathrm {TEI}_i} \end{aligned}$$

where $\mathrm {Food}_{ji}^*$ and $\mathrm {Food}_{ji}$ represent the scaled and unscaled intake of food variable *j* for individual *i*, respectively. The scaling factor divides the total mean intake for all individuals ($\mathrm {\overline{TEI}}$), with the total energy intake for individual *i* ($\mathrm {TEI}_i$).

Although we excluded participants with extreme values of intake, some participants still had remarkably high scaled intake values for some of the food variables. Due to inherent uncertainty of FFQ data on the individual level, it has been recommended that FFQ data are used on group level [23]. For clustering food variables, we therefore chose to divide the study sample in cohorts of approximately 10 participants, resulting in a total of 1,091 cohorts. The partition in cohorts was performed by random sampling of the participants, under the constraint that the cohorts should be homogeneous with respect to background variables. This implied that the participants assigned to each cohort were matched by sex, similar age, educational level (low/high) and PAL (low/high). The intake values for the different food variables were then averaged within each of the cohorts, reducing the effect of extreme and possible erroneous values.

### Statistical analysis

An hierarchical agglomerative clustering method was applied to group similar food variables in non-overlapping clusters. The pairwise distances between the 33 aggregated food variables were measured by Spearman’s rank correlation. The food variables were then merged according to the complete linkage method into clusters. The cluster analysis was repeated for 100 different random samplings of a total of 1091 cohorts. The final diet groups were based on these 100 repetitions, where each food variable was assigned to the diet group in which it occurred most often. This was done to assess the variability of the cluster results and increase the validity of the resulting diet groups [24]. Also, the random sampling of cohorts was used to decide on the number of clusters giving the most stable classification result.

After establishing dietary patterns by cluster analysis, we calculated individual diet intake scores. The score was found by first standardizing the individual scaled intake values ($\mathrm {Food}^{*}_{ji}$) for each food variable *j*. This standardization was done to weight the intake of all food variables equally. For each individual, we then calculated the average of the standardized food variables within each diet group. This made the final intake scores comparable across diets. Note that these individual diet intake scores reflect diet preferences in the sense that larger values imply larger intake of the specific diet. Also, the sum over all individual scores within each diets is 0.

The scores for each diet were then modelled as the dependent variable in regression models on age, including educational level (low/high) and PAL (low/high) as categorical covariates. The association between the diet scores and age was modelled either using a linear or non-linear trend, in which the non-linear trend was modelled using natural cubic splines. Different models were evaluated using the adjusted coefficient of determination ($R^2_{\text{ adj }}$). Subsequent analyses included analysis of covariance and post hoc pairwise testing of diet scores between groups having different combinations of the two categorical variables. Specifically, this included comparison of groups characterized by having low education and low PAL ($\mathrm {L_{edu}L_{PAL}}$), low education and high PAL ($\mathrm {L_{edu}H_{PAL}}$), high education and low PAL ($\mathrm {H_{edu}L_{PAL}}$), and high education and high PAL ($\mathrm {H_{edu}H_{PAL}}$). The analysis was performed separately for men and women and p-values were adjusted using Tukey’s method, ensuring a global significance level of 0.05.

## Results

### Characteristics of the study sample and food intake

The study sample consisted of 5807 women and 5092 men (Table 1). Slightly more than half (53.1%) had high education. This percentage was significantly different for the age groups below and above 60 years, for both women and men ($p < 0.001$ in both). In total $28.3\%$ reported high PAL. The percentage of high PAL was lower in women ($22.3\%$) compared to men ($35.1\%$, $p < 0.001$). Also, the percentage of high PAL decreased with age for both sexes ($p < 0.001$ in both).

**Table 1:** Descriptive statistics for age, educational level and physical activity level (PAL), by sex

			All	Women	Men
N			10899	5807	5092
Age (sd)			57.1 (10.7)	56.5 (10.5)	57.9 (10.9)
Educational level, n(%):					
	Low ($\mathrm {L_{edu}}$)		5114 ($46.9\%$)	2649 ($45.6\%$)	2465 ($48.4\%$)
	High ($\mathrm {H_{edu}}$)		5785 ($53.1\%$)	3158 ($54.4\%$)	2627 ($51.6\%$)
		$40-59$ years old		2302 ($64.9\%$)	1600 ($56.8\%$)
		60 years old and above		856 ($37.9\%$)	1027 ($41.5 \%$)
PAL, n(%):					
	Low ($\mathrm {L_{PAL}}$)		7818 ($71.7\%$)	4511 ($77.7\%$)	3307 ($64.9\%$)
	High ($\mathrm {H_{PAL}}$)		3081 ($28.3\%$)	1296 ($22.3\%$)	1785 ($35.1\%$)
		$40-59$ years old		923 ($26.0\%$)	1120 ($39.8\%$)
		60 years old and above		373 ($16.5\%$)	665 ($29.2 \%$)
Education and PAL groups, n (%):					
	$\mathrm {L_{edu}L_{PAL}}$		3971 ($36.4\%$)	2234 ($38.5\%$)	1737 ($34.1\%$)
	$\mathrm {L_{edu}H_{PAL}}$		1143 ($10.5\%$)	415 ($7.1\%$)	728 ($14.3\%$)
	$\mathrm {H_{edu}L_{PAL}}$		3847 ($35.3\%$)	2277 ($39.2\%$)	1570 ($30.8\%$)
	$\mathrm {H_{edu}H_{PAL}}$		1938 ($17.8\%$)	881 ($15.2\%$)	1057 ($20.8\%$)

The 33 aggregated food variables comprised 7 beverage variables and 26 variables representing solid food items (see Table S2, Additional file 2). Most of the beverage variables were recorded with a maximum intake of more than 3000 g/day, and the largest mean and median scaled intake values were reported for Coffee, Water and Milk. Among the solid food variables, the highest mean and median values of intake were seen for Vegetables, Fruit, Bread, Meat Dinner and Potato, followed by Fish Dinner and Composite Dinner Dishes (in decreasing order). Almost all of the respondents (98-99%) reported an intake for these variables, while about 50% - 60% reported an intake of food variables like Mayonnaise and Plant-based Oils, Chips, Candy and Breakfast Cereals.

### Identification of diet groups by cluster analysis

The cluster analyses identified three main diet groups (Table 2). These dietary patterns are referred to as: The Meat and Sweets diet, including Composite Dinner Dishes, Meat-spread, Meat Dinner, and a variety of sweets; The Traditional diet, including Bread, Fish-spread, Fish Dinner, Milk and Potato; The Plant-based- and Tea diet, including Cereals, Fruit, Nuts, Tea, Vegetables and dairy products.

The given dietary patterns were based on observing the number of times each food variable was clustered to each of the three diet groups, choosing the diet group having the maximum frequency of the 100 random samplings (see Table S3, Additional file 2). A total of five food variables (Beverages with Alcohol, Butter and Margarine, Egg, Juice and Water) were not clearly classified to one of the given three diet groups as these variables frequently switched between clusters in repeated analyses. All but four of the food variables (Beverages with Alcohol, Butter and Margarine, Milk/sugar for Coffee/tea and Water) were clustered to the same diet group for men and women.

**Table 2:** Diet groups from cluster analysis of food variables

	Meat and Sweets	Traditional	Plant-based- and Tea	Inconclusive*
All	Candy, Chips, Chocolate, Soft Drinks, Composite Dinner Dishes, Meat-spread, Mayonnaise and Plant-based Oils, Meat Dinner, Rice/pasta, Sauce etc.	Bread, Cakes and Pastries, Breakfast Cereals (Sweetened), Coffee, Dessert, Fish Dinner, Fish-spread , Milk, Potato, Jam	Cheese, Breakfast Cereals and Porridge (Unsweetened), Fruit, Nuts, Tea, Vegetables, Yoghurt	Egg, Juice
Only men	Water	Milk/sugar for Coffee/tea		Beverages with Alcohol, Butter and Margarine
Only women		Butter and Margarine	Beverages with Alcohol, Milk/sugar for Coffee/tea	Water

* Inconclusive include variables not clearly defined to the either of the three main diet groups

### Identifying dietary patterns across age, level of education and PAL

To make the diet scores comparable for men and women in subsequent analysis, these were calculated using only the food variables classified to the same diet group for both sexes. The average scores for the Meat and Sweets diet were approximately zero for both women ($\text{ sd }=0.42$) and men ($\text{ sd }=0.39$). For the Traditional diet, the average score for women was equal to $-0.05$ ($\text{ sd }=0.33$). The corresponding score for men was 0.05 ($\text{ sd }=0.34$), giving a significant difference between the sexes ($p < 0.001$). Women had a higher intake of the Plant-based- and Tea diet with an average score of 0.13 ($\text{ sd }=0.48$), while the corresponding score was equal to $-0.22$ ($\text{ sd }=0.37$) for men ($p < 0.001$). The correlation between the scores for the Meat and Sweets diet and the Traditional diet was $-0.36$. The corresponding correlations in scores for the Plant-based- and Tea- diet, compared with the other two diets, were both $-0.27$.

**Fig. 2:**
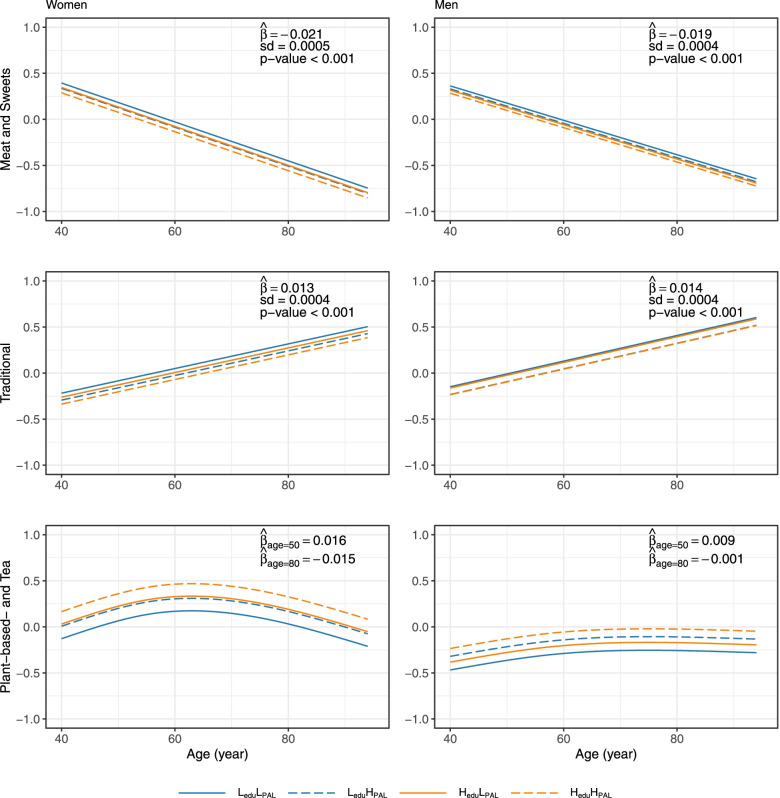
Diet scores for each of the three dietary patterns from the cluster analysis, by age. Estimated trends of diet scores as a function of age, based on data from 5807 women (left panels) and 5092 men (right panels). Four lines are included, where educational level is low(blue)/high (orange) and PAL is low(unbroken lines)/high (dashed lines). For the linear models, the figures include the estimated slopes ($\hat{\beta }$), standard deviations (sd) and the p-value evaluating whether the slope is significantly different from 0. For the non-linear models, the figures include the slope estimates at the age of 50 and 80

For both women and men, the diet scores for the Meat and Sweets diet showed a significantly decreasing linear trend as function of age ($p<0.001$), see Fig. 2. In contrast, the scores for the Traditional diet were observed to have a significant positive linear trend as function of age ($p<0.001$). For the Plant-based- and Tea diet, we chose to model the association between the diet scores and age non-linearly, as this gave a clear relative increase in the adjusted coefficient of determination for women (see Table S4, Additional file 2). For women, the estimated non-linear functions were seen to increase until the approximate age of 60, and then decrease for higher ages. For men, the average score for the Plant-based- and Tea diet increased slightly with age.

The given results were calculated without including interaction terms in the regression models as these did not increase the adjusted coefficient of determination for the different models (see Table S4, Additional File 2). Specifically, interaction between education and PAL was non-significant in all cases. Thus, the estimated linear and non-linear curves of diet scores as function of age were parallel for the four groups $\mathrm {L_{edu}L_{PAL}}$, $\mathrm {L_{edu}H_{PAL}}$, $\mathrm {H_{edu}L_{PAL}}$ and $\mathrm {H_{edu}H_{PAL}}$. However, the mean levels of these curves were shifted vertically, indicating potential significant differences between these groups in terms of the intakes of the different diets.

To further investigate differences in dietary preferences between groups, we performed four pairwise hypotheses tests for differences in the mean diet score. This was repeated for all three diets and both sexes, resulting in a total of 24 tests (Table 3). The group with lower education ($\mathrm {L_{edu}}$) showed a significantly higher intake of the Meat and Sweets and the Traditional diets than those with high education ($\mathrm {H_{edu}}$), and also a significantly lower intake of the Plant-based- and Tea diet for both sexes. Similar results were found in comparing the groups with low ($\mathrm {L_{PAL}}$) versus high PAL ($\mathrm {H_{PAL}}$), except that no significant difference was found for the Traditional diet among men. Naturally, these differences in diet preferences are most clear in comparing the groups $\mathrm {L_{edu}L_{PAL}}$ with $\mathrm {H_{edu}H_{PAL}}$. Among men, the group $\mathrm {L_{edu}H_{PAL}}$ had a significant higher intake of the Traditional diet and a significant lower intake of the Plant-based- and Tea diet, than $\mathrm {H_{edu}L_{PAL}}$.

**Table 3:** Comparison of differences (sd) in diet score between education (low/high) and PAL groups (low/high)

	Meat and sweets		Traditional		Plant-based- and Tea	
Comparisons	Women	Men	Women	Men	Women	Men
$\mathrm {L_{edu} - H_{edu}}$	$0.06*$ (0.01)	$0.03*$ (0.01)	$0.08*$ (0.01)	$0.08*$ (0.01)	$-0.14*$ (0.01)	$-0.15*$ (0.01)
$\mathrm {L_{PAL} - H_{PAL}}$	$0.05*$ (0.01)	$0.05*$ (0.01)	$0.04*$ (0.01)	0.01 (0.01)	$-0.16*$ (0.01)	$-0.09*$ (0.01)
$\mathrm {L_{edu}L_{PAL} - H_{edu}H_{PAL}}$	$0.11*$ (0.01)	$0.08*$ (0.01)	$0.12*$ (0.01)	$0.09*$ (0.01)	$-0.29*$ (0.02)	$-0.23*$ (0.01)
$\mathrm {L_{edu}H_{PAL} - H_{edu}L_{PAL}}$	0.01 (0.02)	$-0.01$ (0.01)	0.03 (0.01)	$0.07*$ (0.01)	0.02 (0.02)	$-0.06*$ (0.02)

* $p<0.05$ by Tukey’s method

## Discussion

The given cross-sectional study in a general Nordic population identified three diet groups, referred to as the Meat and Sweets diet, the Traditional diet, and the Plant-based- and Tea diet. In both men and women, the diet score reflecting the intake of the Meat and Sweets diet showed a significant negative trend with increasing age. The opposite trend was seen for the Traditional diet, in which the scaled intake increased as a function of age. Women had a lower consumption of the Traditional diet and a higher consumption of the Plant-based- and Tea diet than men. Diet preferences were significantly dependent on education level and PAL. Participants with high education had lower diet scores for the Meet and Sweets and the Traditional diets and higher scores for the Plant-based- and Tea diet compared to participants with low education. Participants with high PAL had lower diet scores of the Meat and Sweets diet and the Traditional diet (only women), and higher diet scores of the Plant-based- and Tea diet, compared to participants with low PAL.

The three diet groups identified in this population have similarities to the western, traditional and prudent diets [17, 18, 25]. The Meat and Sweets diet group is similar to the western dietary pattern, characterized by high intakes of red and processed meat (here represented by Composite Dinner Dishes, Meat-spread and Meat Dinner), high intake of sugar drinks (here Soft Drinks) and sweets like Candy and Chocolate. The derived Traditional diet was similar to traditional patterns found in Scandinavia and contained food variables like Bread, Fish-spread, Fish Dinner, Milk and Potato [11, 12, 25]. The Plant-based- and Tea diet group contained some of the food variables being characteristic of a prudent or healthy dietary pattern, including Fruit, Nuts and Vegetables. The similarities in the clustering results for men and women are in accordance with other studies [12, 17].

The age-related differences in diet preferences are in accordance with previous findings where a western diet has shown to be more common among younger ages [17, 26, 27], while more traditional- and prudent diet scores either increase with age or no significant association is found [11, 17, 26–28]. Unless younger individuals gradually change their diet to consuming more traditional foods as they grow older, these findings imply a transition from the Traditional to the Meat and Sweets dietary pattern in the population. However, in this study, we only have data for the population above 40 years of age. Unfavourable developments of diet habits have been observed in the Nordic population between 2011 and 2014, with a decrease in the consumption of fish and whole grains [29]. On the other hand, changes to more healthy dietary patterns by increasing age have been observed for groups with higher education [8–10]. The nutrition transition hypothesis of global dietary convergence to a western diet has been tested and rejected in a study by Azzam [30] over the period 1993-2013, where some countries changed from having a low Western Diet Index (traditional diet) to a high Western Diet Index, and countries previously characterized as having a high Western Diet Index had declined towards a traditional diet during the same period. National health policies on speeding up a transition to healthier dietary patterns in countries with high Western Diet Index, may explain this phenomenon.

The observed dependence between dietary preference and educational level is in accordance with the findings in Nilsen et al. [31]. In this population, a previous study found that participants with higher education had higher odds of nutrition intake in accordance with the Nordic nutrition recommendations [31]. Similar trends have been observed in other populations. For instance, in a study of dietary patterns in Australians aged 55-65 years, high consumption of red- and processed meet and refined grains were associated with low education and low PAL [32]. On the other hand, for different ethnic groups in Netherlands, lower education was not necessarily associated with a significant poorer diet [33].

In studies on food prices and socioeconomic status (SES), Rydén and Hagfors [34] found that healthy diets are more expensive than unhealthy diets in Sweden. Further, children of parents with low education and manual low-skill occupations had the cheapest and most unhealthy diets. It has also been observed that tax and subsidies on unhealthy and healthy food, respectively, improves healthy eating in lower SES [35]. In a Norwegian study on vegetables and fish [36], persons with lower education reported less knowledge in how to prepare these food items compared with persons with higher education. Also, persons with higher education ate significantly more vegetables and fish than persons with lower education. Other findings of the role of education on diet choices are reviewed by Pampel et al. [37]. They summarize research on how problem-solving skills from schooling help making healthier choices and other related questions.

In a German study [38], adults with lower education reported that they consumed energy-dense foods more frequently then adults with higher education. Higher levels of physical work activity among adults with lower education may partly explain why they consume more energy-dense foods. This can also be the case for persons who are physically active in their leisure time. On the other hand, these individuals can be more health-conscious, a factor that also affects food choice. Joo et al. [39] found indication of exercise as a motivation for healthier diets among young adults. Dependent on type of exercise, it was observed that exercise reduced the preference for the western diet and increased the preference for the prudent pattern.

Major strengths of the given analyses include the high Tromsø7 study attendance, and the use of an extensive FFQ to capture the full habitual diet. Further, the diet preferences between groups are investigated using age as a continuous variable. Analyses of diet preferences are often performed by stratifying participants in terms of age groups. This might result in loss of information as the influence of age is assumed constant for participants within a certain age span, e.g. 10 years [40].

Collection of self-reported food intake by FFQs is influenced by several sources of error, including missing data. In the given study, missing data were originally registered as zero, making it impossible to separate zero-intake values from missing data. This limitation of the data material was to a large degree reduced by excluding participants answering less than 90% of the FFQ. The most extreme intake values were excluded in accordance with Lundblad et al. [22], taking into account that intake depend on sex, PAL and body composition.

## Conclusions

The given cluster analysis identified three dietary patterns, referred to as the Meat and Sweets diet, the Traditional diet, and the Plant-based- and Tea diet. Preference for the Meat and Sweets diet and the Traditional diet showed significant negative and positive trends as a function of age, respectively. Adjusting for age, a more unhealthy dietary pattern was associated with low educational level and low PAL.

## Supplementary Information

**Additional file 1:** Appendix.

**Additional file 2:** Table S1-S4.

## Data Availability

The dataset analysed in the current study are obtained from a third party (the Tromsø Study) and are not publicly available. There are legal restrictions set on data availability in order to control for data sharing, including publication of datasets with the potential of reverse identification of de-identified sensitive study participant information. The data can, however, be made available from the Tromsø Study for bona fide researchers upon application to the Tromsø Study Data and Publication Committee. Contact information: The Tromsø Study, Department of Community Medicine, Faculty of Health Sciences, UiT The Arctic University of Norway; e-mail: tromsous@uit.no. Detailed instructions on how to apply are given at the webpage of the Tromsø Study https://uit.no/research/tromsostudy.

## Abbreviations

Tromsø7: The seventh survey of the Tromsø Study
FFQ: Food frequency questionnaire;
TEI: Total energy intake
TWI: Total water intake
PAL: Physical activity level
$\mathrm {L_{edu}L_{PAL}}$: low education and low PAL
$\mathrm {L_{edu}H_{PAL}}$: low education and high PAL
$\mathrm {H_{edu}L_{PAL}}$: high education and low PAL
$\mathrm {H_{edu}H_{PAL}}$: high education and high PAL
KJ: Kilojoule
g: gram
KBS: Kostberegningssystemet (food composition database and nutrient calculation system)
SES: Socioeconomic status
