# Supplementary material for: Identifying dietary patterns across age, educational level and physical activity level in a cross-sectional study: the Tromsø Study 2015 - 2016
Source: BMC Nutr. 2022 Sep 15;8:102. doi: 10.1186/s40795-022-00599-4 (PMC9476603; doi:10.1186/s40795-022-00599-4)
Supplement: Supplementary file 1 — Additional file 1: Appendix. [file 40795_2022_599_MOESM1_ESM.pdf]

Appendix

As over- or underreporting of total food intake is common [1], we chose to exclude data showing extreme intake values. Several methods have been suggested to identify such extreme intakes [4, 2, 3]. Here, we fitted regression models to identify extreme absolute values of residuals. Specifically, we used the total intake values, TEI and TWI, as response variables in separate regression models, as these typically reflect extreme values of the individual food and beverage intake, respectively. These variables were log-transformed to give approximate Gaussian distributions.

The variables TEI and TWI have been seen to depend on body size and composition, PAL, sex and age. We therefore modelled TEI and TWI using height, weight, PAL and age as explanatory variables in the regression models, in addition to sex. Missing values of height and weight were imputed using their respective sex-specific average values. The regression models were fitted using ordinary least squares, providing estimates for the residuals. For each of the regression models, we excluded all cases corresponding to the two percent highest absolute values of the residuals, resulting in 400 participants having an unrealistic food intake.

References

[1] M.H. Carlsen, I.T.L. Lillegaard, A. Karlsen, R. Blomhoff, C.A. Drevon, and L.F. Andersen. "Evaluation of energy and dietary intake estimates from a food frequency questionnaire using independent energy expenditure measurement and weighed food records". In: *Nutrition Journal* 9.1 (2010), p. 37. DOI: 10.1186/1475-2891-9-37.

[2] G.R. Goldberg, A.E. Black, S.A. Jebb, T.J. Cole, P.R. Murgatroyd, W.A. Coward, and A.M. Prentice. "Critical evaluation of energy intake data using fundamental principles of energy physiology: 1. Derivation of cut-off limits to identify under-recording". In: *European journal of clinical nutrition* 45.12 (1991), pp. 569–581.

[3] Marie W. Lundblad, Lene Frost Andersen, Bjarne K. Jacobsen, Monica Hauger Carlsen, Anette Hjartåker, Sameline Grimsgaard, and Laila A. Hopstock. "Energy and nutrient intakes in relation to National Nutrition Recommendations in a Norwegian population-based sample: the Tromsø Study 2015–16". In: *Food & Nutrition Research* 63.0 (2019). ISSN: 1654-661X. DOI: 10.29219/fnr.v63.3616.

[4] J.J. Rhee, L. Sampson, E. Cho, M.D. Hughes, F.B. Hu, and W.C. Willett. "Comparison of Methods to Account for Implausible Reporting of Energy Intake in Epidemiologic Studies". In: *American Journal of Epidemiology* 181.4 (2014), pp. 225–233. DOI: 10.1093/aje/kwu308.
