# Supplementary material for: Identifying dietary patterns across age, educational level and physical activity level in a cross-sectional study: the Tromsø Study 2015 - 2016
Source: BMC Nutr. 2022 Sep 15;8:102. doi: 10.1186/s40795-022-00599-4 (PMC9476603; doi:10.1186/s40795-022-00599-4)
Supplement: Supplementary file 2 — Additional file 2: Table S1-S4. [file 40795_2022_599_MOESM2_ESM.pdf]

Table S1-S4

Table S1: The 244 food items included in the FFQ, aggregated into 33 new food variables plus one variable not used in this study. The food variables represent groups of solid food and beverages.

Groups	Variables
Bread (nr. 1)	FOOD_BREAD_WHITE_T7, FOOD_BREAD_WHOLEGRAIN50_T7, FOOD_BREAD_WHOLEGRAIN100_T7, FOOD_CRISPBREAD_WHITE_T7, FOOD_CRISPBREAD_WHOLEGRAIN_T7
Butter and Margarine (nr. 2)	FOOD_BUTTER_B_T7, FOOD_MARGARINE_BREMYKT_B_T7, FOOD_MARGARINE_BRELETT_B_T7, FOOD_MARGARINE_SOFT_SFSE_B_T7, FOOD_MARGARINE_VITA_B_T7, FOOD_MARGARINE_SFV_ LIGHT_B_T7, FOOD_MARGARINE_MELANGE_B_T7, FOOD_MARGARINE_OTHER_B_T7
Mayonnaise and Plant-based Oils (nr. 3)	FOOD_OIL_OLIVE_OTHER_B_T7, FOOD_MAYONNAISE_B_T7, FOOD_S_MAYONNAISE_SALAD_T7, FOOD_S_MAYONNAISE_SAL_LIGHT_T7
Cheese (nr. 4)	FOOD_S_CHEESE_WHEY_T7, FOOD_S_CHEESE_WHEY_LIGHT_T7, FOOD_S_CHEESE_WHITE_T7, FOOD_S_CHEESE_WHITE_LIGHT_T7, FOOD_S_CHEESE_BLUE_DESSERT_T7, FOOD_S_CHEESE_SOFT_T7, FOOD_S_CHEESE_SOFT_LIGHT_T7, FOOD_S_COTTAGECHEESE_T7
Meat-spread (nr. 5)	FOOD_S_LIVERPASTE_T7, FOOD_S_LIVERPASTE_LIGHT_T7, FOOD_S_SERVELAT_T7, FOOD_S_HAM_BOILED_T7, FOOD_S_SALAMI_T7
Fish-spread (nr. 6)	FOOD_S_CAVIARSPREAD_T7, FOOD_S_CAVIARSPREAD_SVOLVAR_T7, FOOD_S_MACKEREL_TOMATOSAUCE_T7, FOOD_S_SALMON_TROUT_SMOKED_T7, FOOD_S_SARDINES_HERRING_T7, FOOD_S_TUNA_T7, FOOD_S_SCHRIMP_CRAB_T7
Egg (nr. 7)	FOOD_S_EGG_T7
Jam (nr. 8)	FOOD_S_JAM_MARMELADE_T7, FOOD_S_JAM_LIGHT_T7, FOOD_S_PEANUTBUTTER_T7, FOOD_S_CHOCOLATE_NUT_SPREAD_T7, FOOD_S_SWEET_SPREAD_T7

Groups	Variables
Breakfast Cereals and Porridge (Unsweetened) (nr. 9)	FOOD_PORRIDGE_OATMEAL_T7, FOOD_OATMEAL_4GRAIN_T7, FOOD_CEREAL_UNSWEETENED_T7, FOOD_CEREAL_ALLBRAN_T7
Breakfast Cereals - Sweetened (nr. 10)	FOOD_CEREAL_SWEETENED_T7, FOOD_CORNFLAKES_T7, FOOD_CEREAL_HONEY_T7, FOOD_CEREAL_PUFFED_RICE_OAT_T7, FOOD_JAM_CEREAL_T7, FOOD_SUGAR_CEREAL_T7
Milk (nr. 11)	FOOD_MILK_WHOLE_T7, FOOD_MILK_SEMISKIMMED_T7, FOOD_MILK_EXTRASEMISKIMMED_T7, FOOD_MILK_SKIMMED_T7, FOOD_MILK_ BIOLA_CULTURA_NA_T7, FOOD_MILK_ BIOLA_CULTURA_FL_T7, FOOD_MILK_FL_T7, FOOD_HOTCHOCOLATE_T7
Yoghurt (nr. 12)	FOOD_YOGHURT_DRINK_T7, FOOD_YOGHURT_NATURAL_T7, FOOD_YOGHURT_FRUIT_T7, FOOD_YOGHURT_GOMORGEN_MUSLI_T7, FOOD_YOGHURT_LIGHT_FRUIT_T7, FOOD_YOGHURT_LIGHT_MUSLI_T7
Water (nr. 13)	FOOD_WATER_TAP_T7, FOOD_WATER_BOTTLE_T7
Juice (nr. 14)	FOOD_JUICE_ORANGE_T7, FOOD_JUICE_APPLE_OTHER_T7, FOOD_NECTAR_APPLE_OTHER_T7
Soft Drinks (nr. 15)	FOOD_SAFT_SUGAR_T7, FOOD_SAFT_ARTIFICIAL_T7, FOOD_SOFTDRINK_SUGAR_T7, FOOD_SOFTDRINK_ARTIFICIAL_T7, FOOD_ICETEA_SUGAR_T7, FOOD_ICETEA_ARTIFICIAL_T7, FOOD_BEER_NONALCOHOLIC_T7
Beverages with Alcohol (nr. 16)	FOOD_BEER_STRONG_PILS_T7, FOOD_BEER_LIGHT_T7, FOOD_CIDER_ALCOPOPS_T7, FOOD_WINE_RED_T7, FOOD_WINE_WHITE_T7, FOOD_WINE_FORTIFIED_T7, FOOD_LIQUOR_T7, FOOD_COCKTAIL_T7
Coffee (nr. 17)	FOOD_COFFEE_BOILED_T7, FOOD_COFFEE_FILTERED_T7, FOOD_COFFEE_INSTANT_T7, FOOD_COFFEE_ESPRESSO_T7, FOOD_COFFEE_LATTE_T7, FOOD_COFFEE_CAPPUCINO_T7
Tea (nr. 18)	FOOD_TEA_BLACK_T7, FOOD_TEA_GREEN_T7, FOOD_TEA_HERBS_T7

Groups	Variables
Meat Dinner (nr. 19)	FOOD_SAUSAGE_REDMEAT_T7, FOOD_SAUSAGE_REDMEAT_LIGHT_T7, FOOD_SAUSAGE_CHICKEN_TURKEY_T7, FOOD_SAUSAGE_HOTDOG_PORK_T7, FOOD_SAUSAGE_HOTDOG_CHICKEN_T7, FOOD_HAMBURGER_WITH_BUN_T7, FOOD_KARBONADEBURGER_T7, FOOD_MEATBALL_MEATLOAF_T7, FOOD_STEW_MINCED_MEAT_T7, FOOD_STEAK_PORK_BEEF_LAMB_T7, FOOD_CHOPS_PORK_BEEF_LAMB_T7, FOOD_ROAST_PORK_BEEF_LAMB_T7, FOOD_ROAST_GAMEMEAT_T7, FOOD_STEW_MEAT_T7, FOOD_STEW_MEAT_LAPSKAUS_T7, FOOD_BACON_T7, FOOD_CHICKEN_GRILLED_T7, FOOD_CHICKEN_FILLET_T7, FOOD_WOK_T7, FOOD_STEW_CHICKEN_T7
Composite Dinner Dishes (nr. 20)	FOOD_TACO_SHELLS_MEAT_SALAD_T7, FOOD_WRAP_TORTILLA_T7, FOOD_KEBAB_T7, FOOD_LASAGNA_MOUSAKKA_T7, FOOD_PIZZA_T7, FOOD_CALZONA_T7, FOOD_PIE_QUICHE_T7, FOOD_SPRINGROLLS_T7, FOOD_PORRIDGE_SOURCREAM_T7, FOOD_PORRIDGE_RICE_MILK_T7, FOOD_PANCAKES_T7, FOOD_SOUP_VEGETABLE_T7, FOOD_DISH_VEGETARIAN_T7, FOOD_NUDLES_INSTANT_T7, FOOD_OMELETTE_T7
Fish Dinner (nr. 21)	FOOD_FISH_BURGER_PUDDING_T7, FOOD_FISH_BALLS_T7, FOOD_FISH_LEAN_BOILED_T7, FOOD_FISH_LEAN_FRIED_T7, FOOD_FISH_STICKS_T7, FOOD_HERRING_FRESH_SMOKED_T7, FOOD_MACKEREL_FRESH_SMOKED_T7, FOOD_SALMON_TROUT_T7, FOOD_STEW_SOUP_FISH_T7, FOOD_FISH_BAKED_GRATIN_T7, FOOD_WOK_SEAFOOD_VEGETABLES_T7, FOOD_SCHRIMP_CRAB_T7
Potato (nr. 22)	FOOD_POTATOES_BOILED_BAKED_T7, FOOD_POTATOES_MASHED_T7, FOOD_POTATOSALAD_MAJONNAISE_T7, FOOD_POTATO_GRATIN_CREAM_T7, FOOD_POTATOES_FRIED_T7, FOOD_FRENCHFRIES_DEEPFRIED_T7, FOOD_FRENCHFRIES_OVENBAKED_T7
Rice/pasta (nr. 23)	FOOD_RICE_T7, FOOD_PASTA_T7, FOOD_HOTDOGBUN_POTATOWRAP_T7

Groups	Variables
Vegetables (nr. 24)	FOOD_CARROT_T7, FOOD_CABBAGE_T7, FOOD_RUTABAGA_T7, FOOD_CAULIFLOWER_T7, FOOD_BROCCOLI_T7, FOOD_BRUSSELSSPROUT_T7, FOOD_ONION_T7, FOOD_LETTUCE_T7, FOOD_BELLPEPPER_T7, FOOD_AVOCADO_T7, FOOD_TOMATO_T7, FOOD_CORN_T7, FOOD_VEGETABLES_MIX_FROZEN_T7, FOOD_SALAD_MIX_T7, FOOD_BEANS_LENTILS_T7, FOOD_S_VEGETABLES_BREAD_T7
Sauce etc. (nr. 25)	FOOD_SAUCE_BROWN_WHITE_T7, FOOD_SAUCE_BEARNAISE_T7, FOOD_BUTTER_MARGARINE_MELT_T7, FOOD_BUTTER_HERB_T7, FOOD_MAYONNAISE_REMOULADE_T7, FOOD_MAYONNAISE_LIGHT_T7, FOOD_SOURCREAM_T7, FOOD_SOURCREAM_LIGHT_T7, FOOD_SOURCREAM_EXTRALIGHT_T7, FOOD_SALADDRESSING_T7, FOOD_SALADDRESSING_LIGHT_T7, FOOD_SALADDRESSING_OIL_T7, FOOD_SOYSAUCE_T7, FOOD_PESTO_T7, FOOD_SALSA_TOMATOSAUCE_T7, FOOD_KETCHUP_T7, FOOD_MUSTARD_T7
Fruit (nr. 26)	FOOD_APPLE_T7, FOOD_PEAR_T7, FOOD_BANANA_T7, FOOD_ORANGE_T7, FOOD_CLEMENTINE_T7, FOOD_GRAPEFRUIT_T7, FOOD_PEACH_NECTARINE_T7, FOOD_KIWI_T7, FOOD_GRAPE_T7, FOOD_MELON_T7, FOOD_STRAWBERRY_T7, FOOD_RASPBERRY_T7, FOOD_BLUEBERRY_T7, FOOD_CLOUDBERRY_T7, FOOD_RAISIN_T7, FOOD_FRUIT_DRIED_T7, FOOD_FRUIT_B_T7, FOOD_FRUIT_HERMETIC_T7, FOOD_FRUITSALAD_T7
Dessert (nr. 27)	FOOD_ICECREAM_T7, FOOD_ICELOLLY_SORBET_T7, FOOD_PUDDING_T7, FOOD_SAUCE_VANILLA_T7, FOOD_CREAM_WHIPPED_T7
Cakes and Pastries (nr. 28)	FOOD_SWEET_BUN_PRETZEL_T7, FOOD_SWEET_ROLL_CUSTARD_T7, FOOD_PASTRY_DANISH_T7, FOOD_MUFFIN_CAKE_NOICING_T7, FOOD_WAFFLE_T7, FOOD_LEFSE_T7, FOOD_CAKE_CHOCOLATE_BROWNIE_T7, FOOD_CAKE_SPONGE_CREAM_T7, FOOD_BISCUIT_SWEET_T7, FOOD_TREAT_SNOWBALL_T7
Chocolate (nr. 29)	FOOD_CHOCOLATE_T7, FOOD_CHOCOLATE_DARK_T7, FOOD_CHOCOLATE_CONFECTIONS_T7
Candy (nr. 30)	FOOD_PASTILLES_SUGARFREE_T7, FOOD_CANDY_LICORICE_OTHER_T7, FOOD_CANDY_MIX_T7
Chips (nr. 31)	FOOD_CHIPS_POTATOE_T7, FOOD_SNACS_SALTY_T7

Groups	Variables
Nuts (nr. 32)	FOOD_PEANUT_CASHEW_T7, FOOD_ALMOND_HAZELNUT_WALNUT_T7, FOOD_FRUIT_NUT_MIX_T7
Supplements (nr. 33)<br><br>Not included in this study	FOOD_SUPPL_CODLIVEROIL_T7, FOOD_SUPPL_CODLIVEROIL_C_T7, FOOD_SUPPL_FISHOIL_OMEGA3_T7, FOOD_SUPPL_SEALOIL_CAPSULA_T7, FOOD_SUPPL_SANASOL_T7, FOOD_SUPPL_BIOVIT_T7, FOOD_SUPPL_MULTIVIT_MINERAL_T7, FOOD_SUPPL_MULTIVIT_TAB_T7, FOOD_SUPPL_IRON_SULFATE_T7, FOOD_SUPPL_IRON_HEME_T7, FOOD_SUPPL_IRON_FERROCHEL_T7, FOOD_SUPPL_IRON_FLORADIX_T7, FOOD_SUPPL_VIT_B_T7, FOOD_SUPPL_VIT_C_T7, FOOD_SUPPL_VIT_D_T7, FOOD_SUPPL_VIT_E_T7, FOOD_SUPPL_FOLATE_T7
Milk/sugar for Coffee/tea (nr. 34)	FOOD_SUGAR_COFFEE_T7, FOOD_SUGAR_TEA_T7, FOOD_SWEETENERS_COFFEE_TEA_T7, FOOD_MILK_CREAM_COFFEE_TEA_T7

Table S2: Summary of individual food intake (g/day) for the final study sample (n = 10899), adjusted for the individual energy intake (J/day).

Food variables	Mean	Median	25% percentile	75% percentile	Max	Consumers (%)
Bread	148	144	105	186	487	99.2
Butter and Margarine	12	8	0	19	214	72.5
Mayonnaise and Plant-based Oils	5	1	0	7	172	51.2
Cheese	36	30	16	49	290	97.2
Meat-spread	16	13	7	21	133	92.9
Fish-spread	23	17	7	32	277	88.7
Egg	18	14	8	24	390	86.6
Jam	15	10	2	20	283	75.5
Breakfast Cereals and Porridge (unsweetened)	25	8	0	33	636	62.6
Breakfast Cereals (Sweetened)	6	3	1	7	110	81.4
Milk	366	309	132	538	3,201	83.8
Yoghurt	41	18	0	57	1,063	74.5
Water	919	709	430	1,078	10,691	98.5
Juice	86	26	0	108	2,481	62.1
Soft Drinks	149	44	0	143	10,018	70.1
Beverages with Alcohol	166	103	36	219	3,172	89.5
Coffee	934	738	424	1,137	12,845	93.8
Tea	176	36	0	208	9,512	62.1
Meat Dinner	125	118	84	159	553	99.4
Composite Dinner Dishes	89	81	50	119	476	98.6
Fish Dinner	96	87	57	125	429	99.2
Potato	100	90	54	134	485	98.8
Rice/pasta	38	27	12	50	780	93.0
Vegetables	221	188	116	288	1,753	99.9
Sauce etc.	28	24	15	36	190	99.4
Fruit	206	174	96	278	1,517	99.3
Dessert	11	7	2	14	324	81.7
Cakes and Pastries	22	17	8	30	208	91.9
Chocolate	8	5	2	10	177	84.4
Candy	6	2	0	7	160	62.1
Chips	4	2	0	6	124	60.9
Nuts	15	8	2	20	178	82.3
Milk/sugar for Coffee/tea	16	0	0	4	1,616	38.6

Table S3: The number of times each of the 33 food variables is categorized to the three diet groups using cluster analysis on 100 random samplings of the cohorts.

Food variables	Women			Men		
	Meat and Sweets	Traditional	Plant-based- and Tea	Meat and Sweets	Traditional	Plant-based- and Tea
Candy	100	0	0	100	0	0
Chips	100	0	0	100	0	0
Chocolate	100	0	0	99	0	1
Soft Drinks’	100	0	0	100	0	0
Composite Dinner Dishes	100	0	0	100	0	0
Rice/pasta	100	0	0	100	0	0
Mayonnaise and Plant-based Oils’	90	8	2	88	2	10
Meat-spread	96	2	2	90	0	10
Meat Dinner	96	2	2	92	1	7
Sauce etc.	96	2	2	92	1	7
Water	17	40	43	75	7	18
Cakes and Pastries	1	94	5	0	90	10
Dessert	1	94	5	0	90	10
Bread	0	100	0	5	90	5
Fish spread	0	90	10	0	69	31
Jam	0	100	0	0	99	1
Coffee	0	100	0	0	98	2
Fish Dinner	0	76	24	0	99	1
Milk	2	93	5	5	92	3
Potato	0	100	0	0	99	1
Breakfast Cereals (sweetened)	2	93	5	1	80	19
Butter and Margarine	19	76	5	52	38	10
Cheese	2	9	89	1	11	88
Breakfast Cereals and Porridge (unsweetened)	4	1	95	2	7	91
Fruit	0	6	94	0	21	79
Nuts	5	1	94	7	4	89
Tea	7	1	92	0	11	89
Vegetables	0	6	94	0	12	88
Yoghurt	2	21	77	0	23	77
Beverages with Alcohol	8	1	91	51	3	46
Milk/sugar for Coffee/tea	14	10	76	0	75	25
Egg	13	39	48	29	43	28
Juice	28	36	36	48	6	46

Table S4: The adjusted coefficient of determination (R^2_{adj}) for different regression models of diet scores. The diet scores were modeled by age, PAL and education, considering both a linear and non-linear association with age and also inclusion of interaction terms between age, PAL and education.

Models	Women			Men		
	Meat and Sweets	Traditional	Plant-based- and Tea	Meat and Sweets	Traditional	Plant-based- and Tea
Linear	0.259	0.237	0.043	0.262	0.232	0.071
Non-linear	0.272	0.238	0.064	0.271	0.232	0.075
Linear with interaction	0.259	0.241	0.050	0.263	0.233	0.071
Non-linear with interaction	0.273	0.243	0.064	0.271	0.234	0.075
